# Thermophotoinduced electron emission from conductive composite based on polytetrafluoroethylene with carbon nanotubes

**DOI:** 10.1038/s41598-025-12418-4

**Published:** 2025-08-14

**Authors:** I. Ye. Galstian, M. Ya. Shevchenko, Ye. A. Tsapko, T. D. Shatnii, O. M. Lisova, E. G. Len

**Affiliations:** 1 Leibniz Institute for Solid State and Materials Research, Helmholtzstrasse 20, 01069 Dresden, Germany; 2 G. V. Kurdyumov Institute for Metal Physics, N.A.S. of Ukraine, 36 Academician Vernadsky Blvd., Kyiv, 03142 Ukraine; 3 Chuiko Institute of Surface Chemistry, N.A.S. of Ukraine, 17 General Naumov Str., Kyiv, 03164 Ukraine; 4 Kyiv Academic University, N.A.S. and M.E.S. of Ukraine, 36 Academician Vernadsky Blvd, Kyiv, 03142 Ukraine

**Keywords:** Polymer–carbon nanocomposites, Structural defects, Work function, Electron emission, Nanoscale materials, Carbon nanotubes and fullerenes, Materials science, Nanoscience and technology, Nanoscale devices, Nanoscale materials, Other nanotechnology, Techniques and instrumentation

## Abstract

This work explores the potential of conductive polymer–carbon nanocomposites, specifically polytetrafluoroethylene with multi-walled carbon nanotubes (PTFE–CNTs), as efficient electron emitters for emission electronics, low-temperature thermionic energy conversion (TEC), sensors, elements of information storage devices, and materials with targeted control of electromagnetic waves’ absorption/reflection. Our main scientific contribution is the demonstration of electron emission from investigated composite PTFE + 10 wt% CNTs at significantly reduced operating temperatures (near 200 °C), much lower than those for pure CNTs and conventional materials for emission electronics and energy. The research combines experimental studies of electron emission under concentrated solar and pulsed laser radiation, structural characterization of samples by electron microscopy, and positron spectroscopy. The last method and contact potential difference method are used to investigate the electronic properties, including charge transfer between composite components, and the work function of the material. Results indicate that electrons can be emitted from both the surface and subsurface (through the polymer layer) of CNTs. The emission current can be enhanced by the Schottky effect due to the electrical fields of adsorbed cations. Additionally, a novel method for tuning the composite’s work function due to irradiation by low-energy electrons is proposed. It opens pathways for targeted control not only of emission performance but other electronic properties of polymer–carbon nanocomposites, including absorption and reflection of electromagnetic waves in a wide frequency range.

**Supplementary Information:**

The online version contains supplementary material available at 10.1038/s41598-025-12418-4.

## Introduction

The special properties of nanostructured materials, in which electronic processes and phenomena are predominantly quantum, have attracted the interest of many research groups. Previous studies show that carbon nanotubes (CNTs) can serve as electric field concentrators and can be effectively used in electrodes for emission electronic devices and photothermal emission energy converters^1–3^. However, to realize such opportunities, it is necessary to conduct thorough studies on the applicability of nanostructures as sources of electron emission and to search for an ideal matrix for such systems.

Polymer materials can increase their functionality if there are ways to influence their electrophysical properties, usually associated with the addition of appropriate nanofillers^4–6^, including CNTs. Many factors, such as the nature of the matrix polymer, the aspect ratio and the actual values of length and diameter of CNTs, the pretreatment (e.g. covalent functionalization, surface coating with a polymer or surfactant), the level of mechanical loading, the processing technology and the presence of a tertiary structure (e.g. compactification) have a decisive influence on the properties of the formed nanocomposites^7–11^.

Various polymer materials can be used as a matrix for CNTs^12,13^. A composite based on polytetrafluoroethylene with carbon nanotubes (PTFE–CNTs), where the CNTs act as an electron acceptor (with some exceptions), is one of the, in which the CNTs act as electron acceptors (with some exceptions), is one of the most studied structures. Studies have also shown the possibility of using carbon nanotubes as a hole-draining layer^14,15^ and a conductive layer for charge transport^16^ in photovoltaic devices. The incorporation of metallic multi-walled CNTs into a polymer matrix opens up broad possibilities for the creation of new electrode materials for vacuum electronics.

Of particular interest are composites in which conductive filler particles in a polymer matrix can form percolation clusters, thereby making the initially dielectric material conductive. Such systems are usually studied far from the percolation transition point. It is important not to lose sight of the most interesting effects, for example, those caused by the self-similarity of the material structure at different scales or the special sensitivity of such systems to the slightest changes in the parameters of contacts between filler particles (for example, carbon nanotubes (CNTs)) under the influence of external factors. The latter opens up the possibility of purposefully controlling the electronic properties of such composites.

PTFE has a wide band gap and low thermal conductivity due to the absence of conduction electrons. The introduction of nanotubes into the amorphous-crystalline structure of PTFE firstly stabilizes it spatially, protects the CNTs from the chemical influence of the environment, and converts the polymer into an electrically conductive state. In addition, the high anisotropic electrical conductivity and the small radius of nanotubes located in the dielectric matrix are important for the effective absorption of electromagnetic waves (EMWs) by converting the energy of longitudinal oscillations of electrons in the tube with the frequency of the external field into thermal energy transferred into the nanocomposite^17–19^. The effects of the composite’s interaction with EMWs are enhanced by the formation of a stochastic conductive network of CNTs in the composite^20^, and the ability to control the state of the contacts between CNTs and between them and the polymer matrix by changing the external conditions opens up the possibility of controlling the ability of the composite material to absorb or reflect EMWs in a wide frequency range. These and many other exceptional properties of nanocomposites containing nanotubes with typical diameters of 10 to 100 nm are because, unlike other composite materials, they have a huge specific surface area of the interface and a high surface-to-volume ratio of the filler phase.

Increasing the concentration of carbon filler in the polymer matrix leads to a transition from a non-conductive to a conductive state at a certain threshold volume fraction of the conductive filler. As reported in several studies^21,22^, volume fractions corresponding to the establishment of a stable percolation network in a carbon-containing polymer composite are usually observed within 5–30 wt% CNTs. Models describing segregated conductive filler–polymer composites have been presented in^23,24^. However, the experimentally observed percolation thresholds in polymer–CNTs composites were lower than those predicted by statistical percolation theory and depended on the degree of homogeneity of the CNTs distribution in the matrix^25–28^. The critical concentration at which the continuous bulk conductive network formed by CNTs turns into a cluster of aggregated CNTs is in the range of 20–25 wt% CNTs. The CNTs aggregation leads to a decrease in the mechanical and electrical properties of the composite. Therefore, in this work, we mainly focused on the study of composites containing 5–20 wt% CNTs in a polytetrafluoroethylene matrix.

Nowadays, a large number of works are devoted to a detailed description of the methods of synthesis of such systems and the study of their mechanical and electrophysical properties^15,20,29^. There are several reviews on the application of conductive and absorptive properties of polymer–carbon nanocomposites. However, the authors of these works were mainly limited to the problems of developing polymers as screens for electromagnetic waves (EMWs)^7,30^.

We present a new view of the scope of such systems as electron emitters for vacuum electronics and low-temperature thermionic energy converters (TECs). We also propose an original method to control the work function (and related electronic properties) of a polymer–carbon composite by exposing it to low-energy electron beams. Finally, we discuss the prospects for the technological implementation of these effects.

## Methodology and samples Preparation

The CNTs used to produce the composites were supplied by TMSpetsmash Ltd. (Kyiv, Ukraine). Multi-walled carbon nanotubes (MWCNTs) synthesized by chemical vapor deposition were used. Previous experiments^31^ showed that MWCNT samples with low bulk density are preferable for use in composites, especially for PTFE–MWCNTs composites. This density was achieved using coprecipitated Fe_2_O_3_MoO_3_-Al_2_O_3_ catalysts containing Aerosil as a pseudo-liquid diluent for nanotube growth^31,32^. Propylene obtained by dehydration of isopropyl alcohol was used as the carbon source. Ash content by weight is 0.8%; specific surface area is 343 m²/g. The tubes were purified from mineral impurities by annealing in air. The mass loss during heating in air at a rate of 10 °C/min was 5% when heated to 588 °C; 10% – 637 °C; 15% – 676 °C. To purify CNTs from other forms of carbon, the synthesized samples were calcined at 650 °C for 1 h (mass loss was > 60%). Carbon nanotubes were identified by electron microscopy using a JEM-100CXII transmission electron microscope (accelerating voltage – 100 kV, resolving power reaches 2.04 Å). Statistical distribution of nanotubes by outer diameter showed that their diameter is in the range of 10–20 nm. Purity of nanotubes, according to TEM data, is 90–95%. Additional information on the conditions of synthesis and certification of samples is given in^33^ and at the manufacturer’s website^34^.

The polymer polytetrafluoroethylene [-CF_2_-CF_2_-]_*n*_ was used as the polymer matrix. The PTFE–CNTs system (with a mass content of multi-walled carbon nanotubes of 5–20 wt%) was prepared by mixing an aqueous PTFE emulsion (type SFN-1, *d* = 1.51 g/cm^3^, mass fraction of dry residue of 55.25%) and an aqueous dispersion of CNTs. The composite was obtained by homogenization in a mixer with a power of 4–7 kW. The powders obtained after drying were pressed at a temperature of 380 °C and a pressure of 5 MPa. A detailed description of the method is given by its authors in^4,33^.

The angular correlation spectra of the annihilation radiation (ACAR) were measured in the angular intervals from 0 to 25 mrad using a standard annihilation spectrometer with a long-slit geometry, an angular resolution of 1.07 mrad, and a background to peak intensity ratio of up to 5%. The radioactive isotope ^22^Na was used as the positron source. The measured spectra of the ACAR were approximated by the least-squares method with the sum of the parabolic contribution (P) from the annihilation of positrons with free electrons and three Gaussian functions – broad (B), medium (M) and narrow (V), which are determined by the contributions from the annihilation of positrons with bound electrons in atoms, in nanopores (for example, in Stone-Wales defects in CNTs) and free volumes in the polymer, respectively. A more detailed description of the methodology can be found in Ref. [^20,35^ and Appendix].

The maximal operating temperature of the sample is 280 °C, and the melting point of the PTFE polymer matrix is 327 °C. Annealing of the samples was carried out at a temperature of 130 °C in a vacuum of 1 × 10^−6^ Pa. Measurement of the work function (surface potential) was carried out by the contact potential difference (CPD) method in the Anderson variant. The work function of the studied materials was determined relative to the (110) or (100) surface of tungsten single crystals, the work function of which has been reliably established^36^. After heating the PTFE + 10 wt% CNTs sample to 130 °C and cooling it down to room temperature, the initial current-voltage characteristic was recorded, which determines the change of the surface potential after the impact of electrons of a certain energy at a beam current of 10^−6^ A. The composition of the residual atmosphere in the chamber was monitored by a mass spectrometer МХ−7304А. Using the Secondary Ion Mass Spectrometry (SIMS) technique, we determined the elemental composition during the bombardment of the surface with indium ions and studied the gas emission from the sample when it was heated to temperatures of 130 °C, 170 °C, and 220°С.

The method of low-energy full-current spectroscopy (FCS) was used to evaluate the excitation of valence states^36^. It is a variant of the secondary electron spectroscopy method and does not contain undesirable factors associated with the processes of multiple interactions.

The electron emission study was carried out on a solar concentrator (with automatic tracking of the Sun and regulation of the light flux power), including a parabolic mirror with a diameter of 1.5 m, connected to a vacuum chamber, as described in [^37^ and Appendix]. The anode and cathode were in a vacuum of ~ 1 Pa during the irradiation process. The cathode temperature, measured by a thermocouple, was a quantitative characteristic of the solar radiation intensity. The dependence of the emission current (in short-circuit mode) and the voltage at the electrodes (in open-circuit mode) on the cathode temperature was measured, and the current-voltage characteristics were also obtained when an external voltage from 5 to 90 V was applied.

Modeling of the processes of thermophotoemission of electrons under concentrated solar radiation in laboratory conditions was performed using the QUANTUM-15 laser equipped with a quantum-optical generator based on yttrium-aluminum garnet (YAG) with a wavelength of λ = 1.06 μm; pulse duration τ = 5 × 10^−3^ s, pulse energy *E*_L_ = 0.1–1.2 J and spot diameter up to 0.28 cm.

The degree of polymer crystallinity was estimated using X-ray structural analysis methods (X-ray crystallinity). The DRON-UM2 (Bragg-Brentano (Θ−2Θ) scanning in step-by-step scanning mode) was used with X-ray tube 2.5BSV-27 Cu, Beta filter for Cu radiation, water cooling (3 l/min), tube voltage up to 50 kV, tube current up to 50 mA. X-ray pulse count rate error is 0.2%. The obtained data are consistent with the literature data for PTFE^38,39^, but exceed the values obtained by other authors using the differential scanning calorimetry method^40^.

## Experiments and discussion

### Prerequisites for using the polymer with carbon nanotubes as an electron emitter

**Fig. 1:**
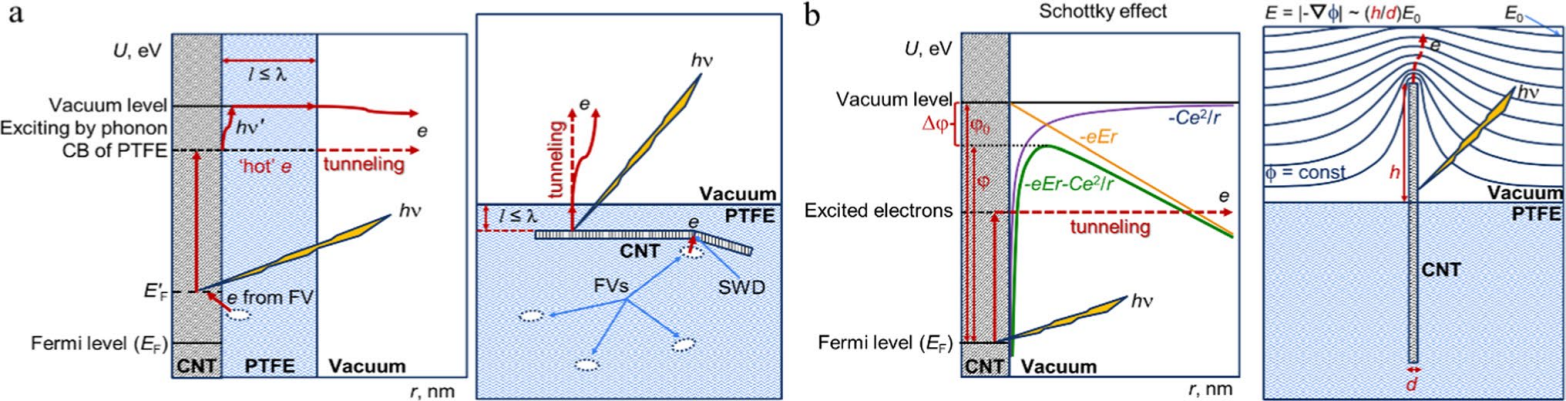
The energy and spatial schemes of electron transfer, thermo- (*h*ν′) and photo- (*h*ν) excitation as well as electron emission from CNT in PTFE matrix, accounting for defects in polymer (free volumes – FVs) and in CNTs (Stone-Wales defect – SWD) (**a**). The analogous schemes illustrated the Schottky effect for CNT in a uniform external electrical field (*E*_0_), which is modified due to the high CNT’s aspect ratio (*h*/*d*) into a nonuniform field (*E*) with a potential (ϕ), and the electron field emission (tunnelling) process in the case of a lowered energy and decreased spatial potential barrier, which determined by the work function φ (φ_0_ is the work function value before the appearance of external electrical field including cases of ions’ adsorption on the sample surface or its irradiation by the electrons) (**b**).

Nowadays, the idea of creating populations of ‘hot’ electrons in dielectric materials for various purposes, which have an energy and a corresponding temperature higher than the temperature of the crystal lattice, is very popular. However, the origins of this idea can be found in the works of the classics, for example, Mott and Gurney in their work^41^, based on the band theory of a solid state, consider the possibility of injecting electrons into an insulator from a corresponding contact (ohmic) in a way almost completely analogous to the injection of electrons from a heated cathode into a vacuum. In other words, electrons ‘evaporate’ from the metal into the conduction band (CB) of the insulator, just as they ‘evaporate’ from a heated cathode into a vacuum. The surface energy barrier of the double layer at the contact of the metal with the insulator can be significantly lower than the corresponding barrier φ (work function) that the electron must overcome to escape from the metal into a vacuum (see Fig. 1). As a result, even at temperatures not exceeding room temperature, the contact can supply a sufficient number of electrons to maintain a current limited by the volume charge (CLVC) in the insulator, which is of exceptional interest from a practical point of view. The transfer of conduction electrons in an insulator (on the path and time of thermalization) is significantly affected by their scattering both on thermal vibrations (phonons) and on chemical (impurity) and structural defects of the crystal lattice. Therefore, the mathematical description of the CLVC in an insulator will differ somewhat from the description of the CLVC in a vacuum. The most significant influence is exerted by local electronic states in the insulator associated with impurities and structural defects that are inevitably present in real materials. Illuminating a sample while maintaining a monopolar carrier injection current can lead to an increase in CLVC if some of the bulk charge is trapped and the trapped carriers can receive energy from the incident light. The last condition can be satisfied in two ways. The trapped carrier can directly absorb a photon and thereby move into one of the allowed bands. Another possibility is an indirect mechanism when the absorbed photon first creates an exciton, which finally transfers its energy to the trapped carrier.

### Analyses of electronic and defects structures and crystallinity of PTFE–CNTs composites

In polymer–carbon nanocomposites, typically, the polymer matrix is a dielectric; therefore, in CNTs and at the CNT-matrix boundaries, processes of generation of nonequilibrium (‘hot’) electrons and holes in excited states under the influence of external factors, as well as processes of their spatial separation, occur. The first type of these processes is associated with the features of the electronic structure of CNTs and defects in them, and the second – with pores of free volume, which are dynamic (thermally activated) defects of the polymer material. It is known that one of the main factors determining the properties of composites is the interface interaction of polymer macromolecules with the filler^26,42^. The most important condition for the successful combination of the properties of nanotubes and polymers is the most uniform distribution of filler particles in the polymer matrix. However, the tendency of CNTs to aggregate due to their high surface energy and large aspect ratio complicates the task.

#### Positron spectroscopy investigation of electronic and defects structures of PTFE–CNTs composites

Due to the ability of positrons to be captured by imperfections in solids with enhanced electron density, positron spectroscopy is widely used to complexly analyze of structural and electronic properties of nanostructured materials. Some positrons can be captured by nanopores and free volumes in polymer, defects, and interlayer spaces in CNTs, etc., and annihilate with electrons in them. All of these processes allow studying the different types of defects and phase interfaces, and electron density distribution in them^27,35^. It is worth noting that the study of short-lived dynamic defects, such as free volumes in polymer, is currently only possible using positron spectroscopy due to the even shorter lifetime of positronium in such defects.

We used positron spectroscopy (ACAR method) to determine the positron annihilation centers and the local electronic structure of defects in the PTFE–CNTs composite. We tested the filler concentration range from 0 to 20 wt%. All positron spectroscopy methods are integral (statistical) and provide sample-average values of defects’ characteristics. The ACAR method is the only one that allows us to study the integral characteristic of the electronic structure of a conductor. In our case, the conductor is a stochastic conductive network of CNTs. This characteristic is reduced to the dependence of the density of free electrons on one of the components of their quasi-wave vector (perpendicular to the plane of the fixed slit of the detector). Our method allows us to directly study several key parameters related to the electronic structure of the material and defects in it. This includes under what conditions and where charges flow between the components of the composite.

**Fig. 2:**
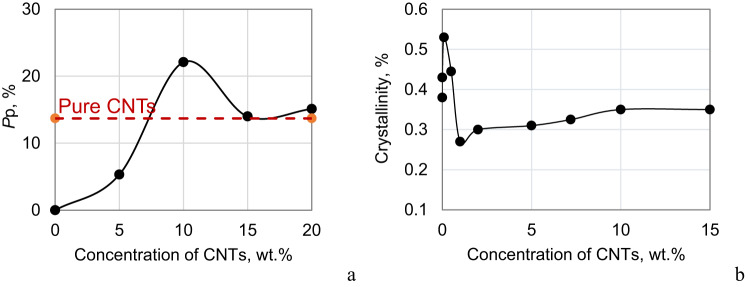
Dependence of the probability of positron annihilation with free electrons (*P*_p_) on the concentration of CNTs in a PTFE-based composite obtained in^20^ by the method of positron annihilation spectroscopy (**a**). Dependence of the degree of crystallinity of the PTFE–CNTs systems on the filler content obtained from the data of X-ray structural analysis (**b**). The diameter of the points on the graphs approximately corresponds to the magnitude of the experimental error.

The ACAR method^20^ yielded clear results: for the samples studied, in the concentration range of up to 5 wt% CNT, the processes of formation of conducting chains in the polymer matrix are already actively underway, and a conducting cluster of multi-walled CNTs has already formed. The probability *P*_P_ of annihilation of positrons with free electrons in the conducting cluster is shown in Fig. 2a^20^. Corresponding concentration dependence of *P*_P_ starts from 0 value for pure PTFE. In the range of 5–10 wt% CNTs, there is a clear increase at the concentration dependence of *P*_P_, which corresponds to the conducting clusters growing, accompanied by electrons flow to CNTs due to the enlargement of the interface PTFE–CNTs area. The curve reaches a peak at 10 wt% CNTs and here, 22% of positrons annihilate with free electrons in CNTs. Then the curve drops sharply to the level of pure CNTs (15–20 wt% CNT), which indicates CNTs’ aggregation in the polymer matrix.

In an ideal polymer matrix, all electrons are covalently bound to their respective atoms, preventing them from undergoing transitions. On the other hand, CNTs contain both bound and free electrons. However, the low free electron density at the Fermi level (*E*_F_) in graphene-like materials (including CNTs) significantly reduces the probability of participation of these electrons in transfer phenomena in the composite, which reduces the probability of emission. It is clear that uncompensated charges and corresponding dipole (multipole) moments in polymer matrix can arise only in the area of defects (molecules buckling) in polymer chains. However, static defects in polymer rarely fall into the areas of Stone-Wales defects (SWDs) in CNTs, through which charges most easily flow from and to CNTs. Conversely, dynamic defects, such as free volumes (FVs), manifest uniformly throughout the polymer volume due to thermal fluctuations. Consequently, these defects often temporarily fall into the areas of defects in CNTs. This is the location and time where electron density can flow and charge can be induced on the CNTs. A direction of charge flow (from the Stone-Wales defect to the polymer matrix or vice versa) depends on whether the average electron density in the Stone-Wales defect or in the polymer free volume is higher or lower (this leads to a shift of Fermi level to a new position *E*_F_; see Fig. 1a). The values of the parameters *S*_B_, *R*_B_ (Table 1) describe the annihilation in Stone-Wales defects and other bound electrons in CNTs and polymer matrix. This allows us to state that the CNTs in the composite contain a fairly large number of Stone-Wales defects with enhanced electron density, so near 21% (*S*_B_/2) of positrons annihilate in them. The parameters *S*_M_, *R*_M_ indicate annihilation in the spaces between both the graphene layers of MWCNTs (not less than 16.5% (*S*_M_/2) of positrons annihilate here) and the layers of the polymer matrix, and *S*_V_, *R*_V_ indicate annihilation in the pores of the free volume in the polymer only. The data in Table 1 related to CNTs is fully confirmed by the X-ray structural analysis presented in^35^.

The analysis of the results shows that in the studied composites with 10 wt% CNTs, the major part of positrons (not less than ~ 60%) annihilate in CNTs, the electronic properties of which are significantly modified by the polymer environment, mainly due to an increase in the electron density on CNTs (for details, see^20^). The conclusion about electron flow from defects of the polymer matrix (from free volumes) through Stone-Wales defects to the CNTs, is confirmed by both (i) the decrease in the probability *P*_P_ of positrons annihilation with (quasi)free π-electrons in MWCNTs with deviation of MWCNTs concentration in the polymer from 10 wt% (Fig. 2a), (ii) the increase in the size of *R*_B_ Stone-Wales defects upon the addition of 10 wt% CNTs to the polymer with the simultaneous increase in the probability of *S*_B_ annihilation of positrons in these defects (Table 1), (iii) the increase in the interplanar distance *R*_M_ in multilayer CNTs in the composite compared to pure MWCNTs due to additional electron density on CNTs, (iv) the decreasing in probability *S*_V_ of positrons annihilation in the pores of free volume in the composite compared with pure PTFE, which indicates a decrease in the electron density in polymer. At the same time, the increase in the size *R*_V_ of free volumes in the composite with 10 wt% CNTs compared to the pure PTFE is consistent with a decrease in the packing density of polymer chains at CNTs concentrations from 1 wt% and higher due to a decrease in the degree of crystallinity of the polymer matrix (Fig. 2b).

**Table 1:** Parameters of the ACAR spectra calculated for cnts, PTFE and the PTFE + 10 wt% CNTs composite: *S*_V_ is the fraction of positrons annihilated in free volumes in PTFE with average radii *R*_V_; *S*_B_, *S*_M_ are the fractions of positrons annihilated with bound electrons in atoms and in nanopores, respectively, and *R*_B_, *R*_M_ are the radii of the corresponding Spatial distributions of electron density in CNTs and polymer.

Samples	S_V_, %	*R*_V_, nm	S_M_, %	*R*_M_, nm	S_B_, %	*R*_B_, nm
CNT	-	-	51.01	0.132	35.30	0.054
PTFE	4.10	1.10	62.33	0.119	33.57	0.055
PTFE + 10 wt% CNTs	3.13	5.14	33.02	0.143	41.76	0.059

#### Crystallinity and nanostructure of PTFE–CNTs composites

As shown in Ref^43^. by the methods of differential thermal analysis and X-ray structural analysis, polytetrafluoroethylene is characterized by a reduced crystallinity of ~ 42%, a significant amount of disordered amorphous phase, and the presence of nanopores. The nanosized filler creates effective crystallization centers in PTFE and therefore even low concentrations of CNTs cause faster crystallization of the polymer.

As demonstrated in Fig. 2b, the data on the degree of crystallinity of the polymer matrix of composites with varying CNTs content indicates that the incorporation of a modest quantity (less than 1 wt%) of multi-walled CNTs into the PTFE matrix results in a substantial enhancement of the crystallinity of the nanocomposite. This effect is manifested in the change of the PTFE lattice parameters, its texture, sizes of coherent scattering blocks, and magnitude of micro stresses, as well as in the increase of crystallization temperature in the PTFE–CNTs system. With increasing filler content, there are too many crystallization centers and they begin to suppress the growth of the crystalline phase, leading to the growth of globules and some increase in the amount of disordered PTFE phase compared to the pure polymer, which, as already noted during the analysis of positron annihilation data, is accompanied by an almost fivefold increase in the size of free volumes. On the one hand, this can be interpreted as the formation of a more defective polymer structure during its amorphization, and on the other hand, it should be considered that amorphization is accompanied by an increase in the interface between the crystalline and amorphous phases with a reduced electron density due to charge flow onto the CNTs. The potential to enhance the degree of crystallinity of PTFE through the incorporation of CNTs (whose crystallinity reaches its maximum at 0.1 wt% CNTs) constitutes a noteworthy finding in its own right. However, the present study does not concern itself with the concentrations of CNTs up to the percolation threshold, as the resultant composites remain dielectrics. At concentrations exceeding 2 wt% CNTs (after attaining the percolation threshold), the degree of crystallinity undergoes a slight increase, reaching a state of saturation at 10 wt% CNTs. The independence of this characteristic from the concentration above 10 wt% CNTs serves as an additional confirmation of the aggregation of CNTs at their high content in the composite, which is completely consistent with the conclusions of positron spectroscopy.

**Fig. 3:**
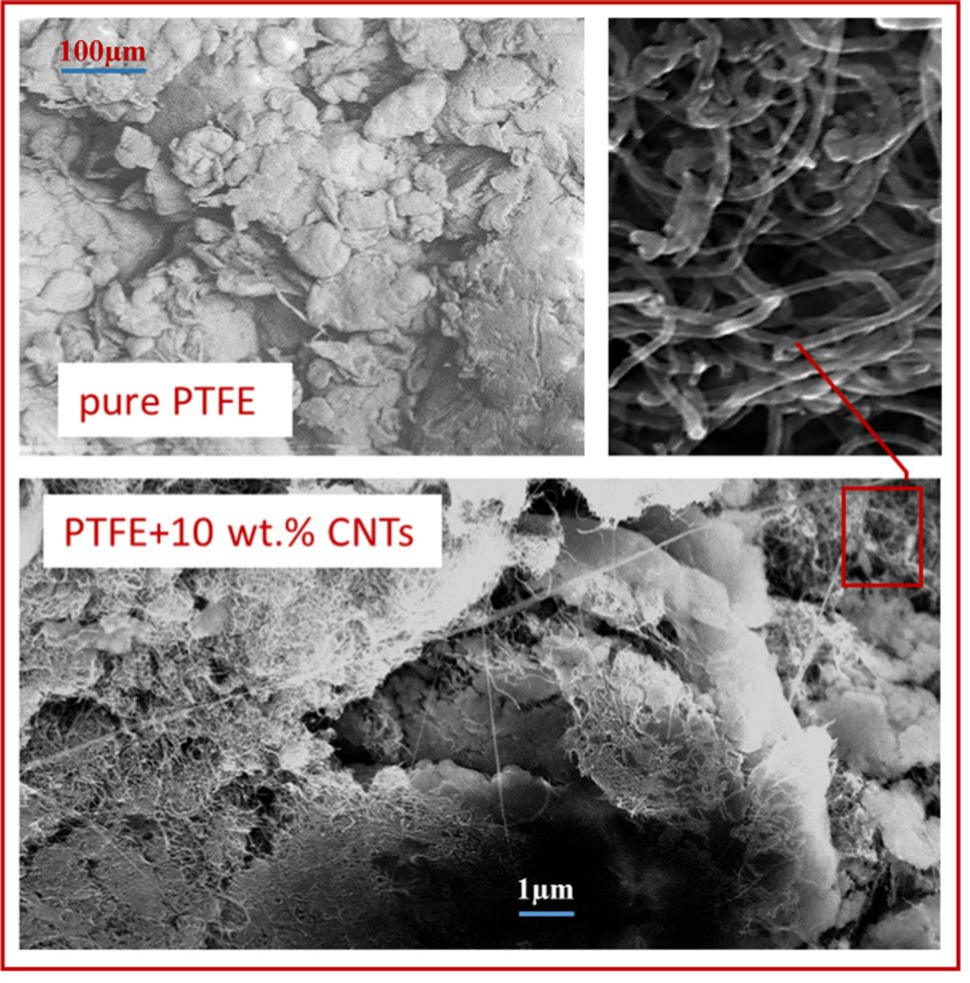
Electron microscope images of the pure PTFE and PTFE + 10 wt% CNTs composite (compilation, original images are given in the Appendix).

Figure 3 shows electron microscope images of the PTFE–CNTs composite for CNTs concentrations of 10 wt%. It can be seen that multi-walled CNTs form a branched network in the polymer matrix (because of the metallic-type of MWCNTs, this network is also electrically conductive). Given that CNTs are enveloped by polymer shells, these images facilitate the estimation of the thickness of the polymer (dielectric) matrix layers that electrons must surmount during their emission. Our estimates indicate that the thickness of polymer layers is approximately 15–20 nm. This is calculated by determining the difference between the average diameter of MWCNTs in a polymer shell (~ 50 nm) and the diameter of pure MWCNTs (10–20 nm), and dividing it by two.

#### Structure of PTFE–CNTs composites and possible mechanism of electron emission

The above-described effects are important from the point of view of the justification of the previously mentioned mechanism of electron emission from a composite cathode with PTFE + 10 wt% CNTs under the action of concentrated sunlight or laser irradiation. First, the maximum number of free electrons in CNTs in this composite (see Fig. 2a) indicates that CNTs in the amount of 10 wt% can be considered as a metallic component of the composite. The presence of free electrons allows the creation of populations of ‘hot’ electrons in CNTs during irradiation of the sample with light. When such metallic particles are near the emitting surface, the CNTs either themselves reach the surface and emit electrons due to the large aspect ratio (Fig. 1b), or border on the vacuum through thin dielectric layers of PTFE (Fig. 1a). The presence of structural defects in CNTs with excess electron density and the disorder of the CNTs’ conductive network can additionally ensure the presence of electron energy levels that will be close to the boundaries of the dielectric band gap, which facilitates the transition of electrons excited in CNTs to the PTFE conduction band. If the thickness (*l*) of the dielectric layer between CNTs and the vacuum is less than the mean free path (λ) of a ‘hot’ electron in the dielectric (see Fig. 1a), such an electron can appear on the emitter surface without losing energy, and then pass into the vacuum absorbing an additional portion of energy from phonons or external photons. Such electrons can also tunnel (if the emitting region of the polymer has a small or negative electron affinity) or they can be captured by cations, which then become neutral atoms that desorb from the surface and transport electrons into the vacuum gap, where these atoms can again be ionized by light (gas discharge conditions).

It should also be noted that the longer path, along which the ‘hot’ electrons thermalize in the dielectric, provides a bigger number of electrons that can reach the surface and leave it. This will increase the emission current. The mean free path depends on the probability of electron scattering on phonons and chemical and structural defects. In this case, the relatively low emitter temperature helps to reduce the probability of electron-phonon collisions, and the presence of large electron-depleted free volumes in the dielectric can help to reduce scattering on structural defects due to electron jumps (tunnelling) between free volumes, the sizes of which are affected by changes in the nanometre region (from 0 to 5 nm). In this study, we have focused on two of the most probable mechanisms affecting the movement of electrons through the polymer matrix. However, it should be noted that changes can be introduced into various established mechanisms of electron transport to the emitting surface.

Thus, the structure of the studied composite with 10 wt% CNTs promotes to increase both the population of ‘hot’ electrons in CNTs and the length of their mean free path in PTFE and, accordingly, the emission current. It is also worth noting that all the characteristics of the ACAR spectra for PTFE–CNTs composites mentioned in this work have extrema at a CNTs concentration of 10 wt%^20^.

In light of the aforementioned considerations, the PTFE–10 wt% CNTs sample was identified as the most promising candidate for subsequent experimentation, with the objective of investigating its emission properties.

### Electron emission from polymer–carbon composites under gas discharge conditions

Let’s consider in more detail the regularities and mechanisms of electron emission for nanostructured materials under gas discharge conditions when the cathode surface is continuously bombarded by positive ions.

Thin tips of nanotubes in the electric field of positive ions or the contact field enhance it by several orders of magnitude, which lowers the potential barrier for the electron (Schottky effect, see Fig. 1b) and reduces the energy losses for the escape (tunneling) of electrons from nanotubes to the adsorbed or nearest positive ion, which, after neutralization, desorbs and transfers the charge from the cathode to the active zone of the interelectrode space, where another ionization occurs with the formation of electrons and positive ions. The process of charge separation occurs: electrons fly to the anode, which has a lower work function, and positive ions, under the influence of the field, fly to the cathode, attaching an electron from the cathode and finally desorbing in a neutral state. Thus, electrons are transferred to the anode with a low work function, and positive ions migrate under the action of the electric field to the cathode with a higher work function (i.e., in cathode energy of free electrons is smaller than in the anode). According to our working hypothesis, the thermionic converter and other vacuum electronic devices work according to 4 stages: (1) absorption of a quantum and ionization under the action of an external ionizer (energy generation and plasma formation); (2) charge separation (adiabatic expansion of the plasma)—electrons are transferred to the anode and positive ions to the cathode, where they are adsorbed on the surface of the CNTs; (3) neutralization of the ions by tunneling of the fastest electrons from the CNTs (which leads to cooling of the cathode); (4) desorption of atoms and transfer of the captured electrons by them.

**Fig. 4:**
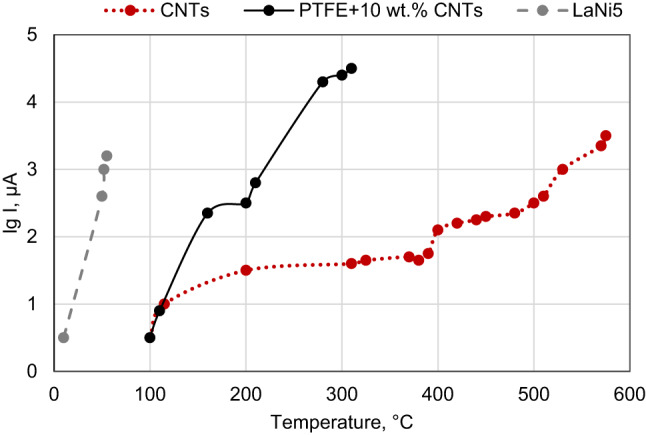
Dependences of the emission current under the influence of concentrated solar radiation on the cathode temperature for the pure CNTs, LaNi_5_ particles with nanotubes on the surface^2^, and PTFE with 10 wt% CNTs.

As part of tests on a solar concentrator (Fig. 4), we found that the temperature of the cathode under solar irradiation heating at which electron emission begins for the PTFE–10 wt% CNTs sample is less than 200°С, which is significantly lower than one for pure nanotubes (near 400°С). Here we only provide an upper estimate of the emission threshold temperature (near 200 °C), since in the region of lower temperatures there are jumps in the temperature dependence of the emission current, which are not described by the well-known Richardson-Dushman equation and indicate the occurrence of more complex processes in the system, which we do not consider here.

It should be noted that thermophotoinduced electron emission at relatively low temperatures (below 200 °C) during solar irradiation was previously observed by us in metal-carbon nanostructures, namely, for a system based on particles of LaNi_5_ with nanotubes on the surface^2^ and for a powder composite based on titanium with the addition of thermally expanded graphite^37^. As can be seen from Fig. 4, polymer matrix-based composites are still inferior in their emission properties to such a well-known cathode material as LaNi_5_^2^. However, unlike metallic and semiconductor materials, polymer–carbon nanocomposites provide greater freedom in simultaneously engaging multiple emission mechanisms, as well as in their adjustment by controlling the composition, volume, and surface structures of the sample. They are also lighter, more affordable, and environmentally friendly.

**Fig. 5:**
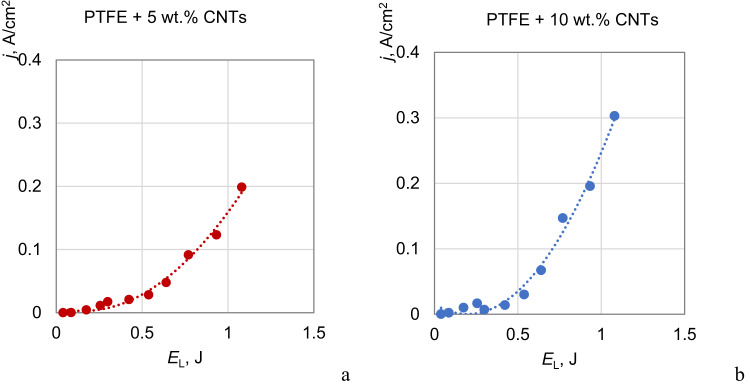
Dependences of the emission current on the laser pulse energy for PTFE + 5 wt% CNTs (**a**) and PTFE + 10 wt% CNTs (**b**).

Modeling of the emission processes using pulse laser radiation confirmed that for PTFE–CNTs composites, the emission current increases with increasing CNT concentration (Fig. 5). For example, at low laser pulse energies (about 1 J), which simulate in laboratory conditions the photothermal emission under solar radiation, the emission current for the PTFE + 5 wt% CNTs sample is 0.2 A/cm^2^, and with the addition of 10 wt% CNTs, the emission current increases by 1.5 time (to 0.3 A/cm^2^).

### The effect of low energy electrons on the work function of the PTFE + 10 wt% CNTs composite

When the sample is heated to 130 °C, a shift of the initial I-V characteristic towards lower voltages is observed. Such a shift indicates a decrease in the negative potential of the surface, which slows down the electrons falling on the sample. This effect indicates a decrease in the work function. The measurements were carried out in a vacuum of 1 × 10^−6^ Pa. The composition of the residual atmosphere was determined mainly by particles with the following masses: 2, 17, 18, 28, 40, and 44 Da.

**Fig. 6:**
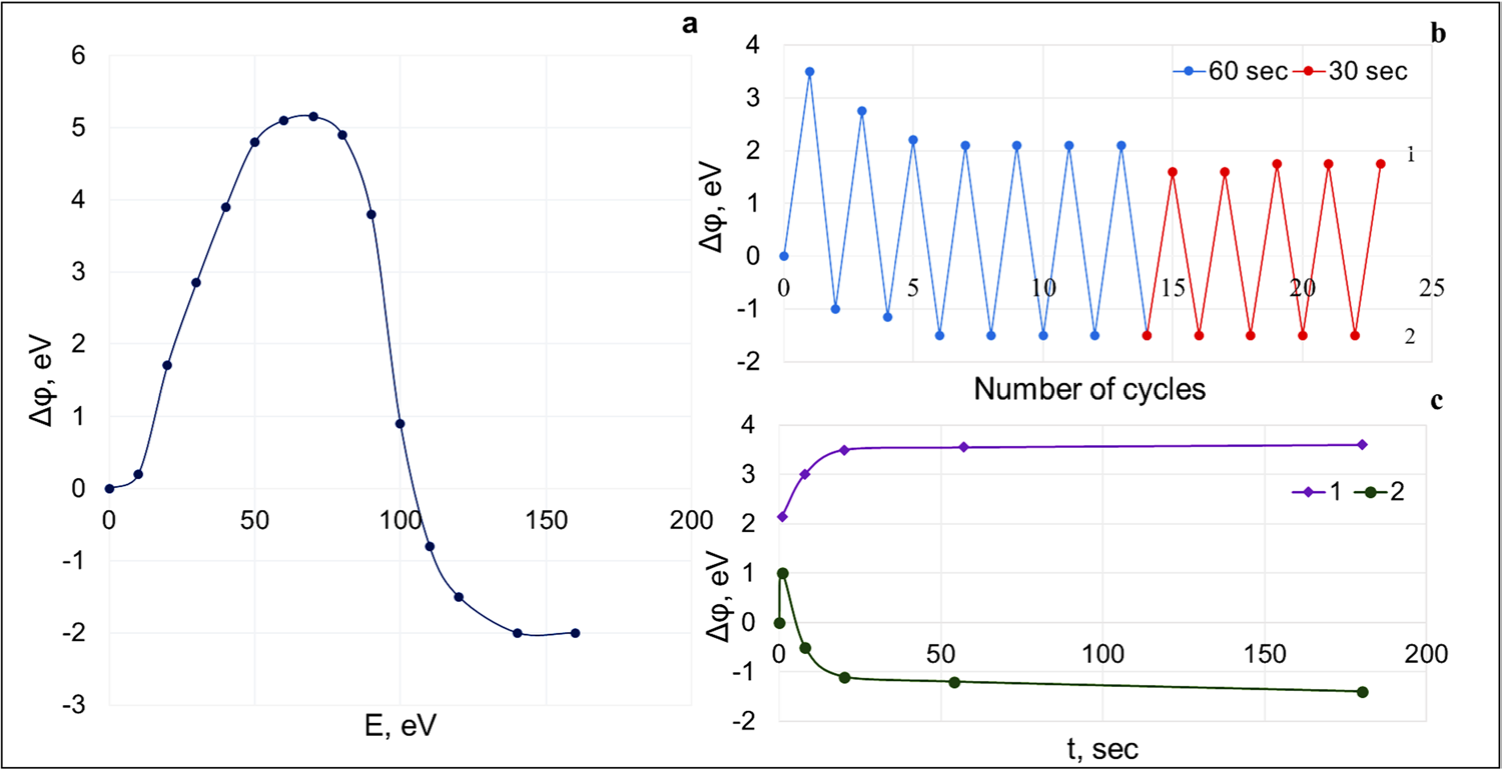
Dependence of the work function changes Δφ after heating the sample at 130 °C (*t* = 4.5 h, electron exposure time 3 min for each energy value *E*) (**a**); work function change Δφ during multiple sequential cyclic sample exposures in the beam of electrons with energies of 60 eV (1) and 120 eV (2) (duration of each cycle is 60 s (I) and 30 s (II)) (**b**); dependence of Δφ(*t*) for *E* = 60 eV (1) and *E* = 120 eV (2) after heating the sample at 130 °C (*t* = 9 h) (**c**).

Figure 6a shows the dependence of the work function change Δφ under the action of electrons after the general heating of the sample at 130 °C for 4.5 h. The time of electron impact at each energy value was 3 min. It can be seen that initially the increase in electron energy *E* leads to the increase in the work function (an increase in the negative surface potential), and the maximum value of the work function increment Δφ = 5.0–5.2 eV occurs at *E* = 60 eV. After that, one can see a decrease in Δφ, and after 110 eV the work function becomes less than the initial value after heating and reaches a minimum at *E* = 140 eV, which corresponds to the negative value Δφ = −2.2 eV.

Figure 6b shows the change of the work function Δφ during multiple sequential cyclic sample exposures in the beam of electrons with the energies of 60 eV (1) and 120 eV (2) during the times of 60 s (I) and 30 s (II). The total heating time was 7 h. After heating the sample at 130 °C for 10 min and cooling it to room temperature, we recorded the initial I-V characteristic. Then, the sample surface was irradiated by electrons with *E* = 60 eV for 60 s (in region I) and the I-V characteristic was recorded. It shifted to the right (Δφ > 0) relative to the initial I-V characteristic. The surface was then irradiated with electrons of energy *E* = 120 eV and the I-V curve was again recorded. Now, it shifted to the left (Δφ < 0) relative to the initial curve. In region II, similar measurements were repeated with an exposure time in the electron beam of 30 s.

After the second cycle of exposure in the electron beam during 60 s, a shift of the I-V characteristic for the two surface states in adjacent cycles is 4.5 V. This shift value multiplied by electron charge *e* determines the work function change Δφ for two selected surface states in Fig. 6. The values of the shift after 13 cycles practically stops changing and is ~ 3.8 V for adjacent cycles (region I in Fig. 6b). When the exposure time is 30 s (region II in Fig. 6b), the similar steady-state value of shift is ~ 3.4 V. Such cyclic exposures were performed with different durations: 1, 5, 10, 20, 30, 40, 60, and 180 s. Analysis of the results shows that the shorter exposure time leads to the smaller change in Δφ values. This is evident from Fig. 6c, which shows the dependence of Δφ(*t*) for *E* = 60 eV (1) and 120 eV (2) for different exposure times after heating the composite at 130 °C for 9 h. If for *E* = 60 eV at an exposure time of *t* = 1 s the value of Δφ = 2.20 eV, and with increasing *t* the function Δφ monotonically increases and reaches saturation (3.80 eV) at 180 s, then for *E* = 120 eV there is a non-monotonic behavior of Δφ with time: the function reaches a maximum of + 0.9 eV at *t* = 1 s, and then decreases, taking negative values (for example, Δφ = −0.48 eV at 10 s) and approaching saturation with Δφ = −1.72 eV at 180 s.

It’s important to remember that an emission electronics device cannot function without additional energy expenditure, for example, a TEC cannot be a source of e.m.f. if the work function of its cathode is lower than the work function of the anode. The value of TEC’s e.m.f. is determined by the difference between the work functions of the cathode and the anode. In our case, it reaches significant values that can be controlled by irradiating the cathode with low-energy electrons.

## Conclusions

For the composite PTFE–CNTs with the CNTs concentration of 10 wt%, significant changes in the parameters of positron annihilation and in the values of the degree of crystallinity of the composite are observed. Higher values of the probability of positron annihilation with free electrons and bound electrons in Stone-Wales defects in CNTs embedded in the composite, in comparison with pure CNTs, indicate that the multi-walled CNTs in the polymer matrix are the electron acceptors.

It was found that under the influence of electromagnetic radiation, ‘heated’ (photoexcited) electron emission from carbon nanotubes immersed in the polymer occurs at lower temperatures and with lower energy losses than in the case of pure CNTs. The electron escape from the conducting CNT’s net through the dielectric layer into the vacuum can be performed in two stages: the transition of the excited electron from the CNT to the conduction band of the dielectric and from the latter to the vacuum.

During modeling the emission process by pulsed laser radiation, we obtained maximal emission currents up to 0.3 A/cm^2^ for cathode made of PTFE + 10 wt% CNTs. In general, the structure of the studied composite promotes an increase in both the population of ‘hot’ electrons in CNTs and their mean free path in PTFE and, accordingly, intensifies the emission current, which we observed on the solar energy concentrator at temperatures starting from 200 °C.

We established a nonlinear dependence of the work function change Δφ of the conductive PTFE + 10 wt% CNTs composite on the energy of the electrons irradiating it during 3 min in the range of energy values of 0-160 eV, which has a maximum at 70 eV and a minimum at 160 eV. The difference between the extreme values of the work function (φ_max_-φ_min_) is more than 7 eV. The work function change Δφ during the sequential cyclic action of electrons leads to a cyclic change of the work function in the range of about 3–4 eV. These results are an important prerequisite for further research of the investigated composite materials in the direction of their electronic properties targeted control.

In addition, it was found that the degree of crystallinity of PTFE increased by adding a small amount of CNTs to the polymer. The maximum crystallinity is observed at 0.1 wt% CNTs. At all values of CNT concentration (less than 1 wt% CNTs), when the crystallinity of the composite is higher than that of pure PTFE, the corresponding composites are dielectrics (are in a state below the percolation threshold).

In conclusion, we describe the areas of possible use of this class of objects. Electron emitters are traditionally used as cathodes in vacuum electronics and power engineering (TEC) devices. Although polymer matrix-based composites still have inferior emission properties compared to well-known cathode materials such as LaNi_5_, they offer greater flexibility in terms of emission mechanisms and structure control; they are also lighter, more affordable, and more environmentally friendly. In addition, non-traditional use of such materials and the effects found in them is also possible, for example, in ultrasensitive sensors of combined heat and light fluxes. It is important that in the systems we study there is no need to adjust the band gap, the very presence of which narrows the spectrum of frequencies available for registration by semiconductor sensors. Irradiation of such systems with electrons allows us to significantly change not only their work function, but also the position of the Fermi level in the energy spectrum of electrons, which opens up one of the ways to control the electronic properties of such composites, for example, the values of their absorption and reflection coefficients of electromagnetic waves. Elements for information storage devices can also be created from such materials.

## Electronic supplementary material

Below is the link to the electronic supplementary material.

Supplementary Material 1

Supplementary Material 2

Supplementary Material 3

## Data Availability

All data collected or analyzed during this study are included in this article and its supplemental information files.

## References

[1] Dai, L. et al. Aligned nanotubes. *ChemPhysChem* 4, 1150–1169 (2003). 10.1002/cphc.200300770 10.1002/cphc.200300770 14652993

[2] Sydorchenko, I. M. et al. Emission properties of cathode materials based on LaNi_5_–CNT composites. *Metallofiz Noveishie Tekhnol.* **43**, 1707–1721. 10.15407/mfint.43.12.1707 (2021).

[3] Nwanno, C. E., Thapa, A., Watt, J., Simkins Bendayan, D. & Li, W. Field emission properties of Cu-filled vertically aligned carbon nanotubes grown directly on thin Cu foils. *Nanomaterials* **14**, 988. 10.3390/nano14110988 (2024). 38869613 10.3390/nano14110988 PMC11174008

[4] Sementsov, Y. I. et al. Properties of PTFE–MWNT composite materials. In *Hydrogen Materials Science and Chemistry of Carbon Nanomaterials. NATO Security Through Science Series A: Chemistry and Biology* (eds Veziroglu, T. N. et al.) 757–763 (Springer, 2007). 10.1007/978-1-4020-5514-0_95.

[5] Das, T. K., Ghosh, P. & Das, N. C. Preparation, development, outcomes, and application versatility of carbon fiber-based polymer composites: A review. *Adv. Compos. Hybrid. Mater.* **2**, 214–233. 10.1007/s42114-018-0072-z (2019).

[6] Andrews, R. & Weisenberger, M. C. Carbon nanotube polymer composites. *Curr. Opin. Solid State Mater. Sci.* **8**, 31–37. 10.1016/j.cossms.2003.10.006 (2004).

[7] Nivedhitha, D. M. & Jeyanthi, S. Polyvinylidene fluoride—An advanced smart polymer for electromagnetic interference shielding applications—A novel review. *Polym. Adv. Technol.* **34**, 1781–1806. 10.1002/pat.6015 (2023).

[8] Khan, W., Sharma, R. & Saini, P. Carbon nanotube-based polymer composites: synthesis, properties and applications in *Carbon Nanotubes - Current Progress of their Polymer Composites* (Eds. Berber M.R. & Hafez I.H.) 1–45 (IntechOpen, 2016). 10.5772/62497

[9] Smith, A. T., LaChance, A. M., Zeng, S., Liu, B. & Sun, L. Synthesis, properties, and applications of graphene oxide/reduced graphene oxide and their nanocomposites. *Nano Materials Science* **1**, 31–47. 10.1016/j.nanoms.2019.02.004 (2019).

[10] Zucolotto, V., Avlyanov, Gregorio, R. J. & Mattoso, L. H. C. Melt processing of composites of PVDF and carbon black modified with conducting polymers. *Appl. Polym.* **94**, 553–557. 10.1002/app.20952 (2004).

[11] Spitalsky, Z., Tasis, D., Papagelis, K. & Galiotis, C. Carbon nanotube-polymer composites: Chemistry, processing, mechanical and electrical properties. *Prog. Polym. Sci.* **35**, 357–401. 10.1016/j.progpolymsci.2009.09.003 (2010).

[12] Alturaif, H. A., ALOthman, Z. A., Shapter, J. G. & Wabaidur, S. M. Use of carbon nanotubes (CNTs) with polymers in solar cells. *Molecules* **19**, 17329–17344. 10.3390/molecules191117329 (2014). 25353384 10.3390/molecules191117329 PMC6271889

[13] Song, K., Zhang, Y. & Minus, M. L. Using low concentrations of nano-carbons to induce polymer self-reinforcement of composites for high-performance applications. *MRS Online Proc. Libr.* **1752**, 137–144. 10.1557/opl.2015.254 (2015).

[14] Kymakis, E., Alexandrou, I. & Amaratunga, G. A. J. High open-circuit voltage photovoltaic devices from carbon-nanotube-polymer composites. *J. Appl. Phys.* **93**, 1764–1768. 10.1063/1.1535231 (2003).

[15] Scardaci, V., Rozhin, A. G., Hennrich, F., Milne, W. I. & Ferrari, A. C. Carbon nanotube–polymer composites for photonic devices. *Phys. E Low-Dimens Syst. Nanostruct.* **37**, 115–118. 10.1016/j.physe.2006.08.001 (2007).

[16] Avouris, P. Carbon nanotube electronics. *Chem. Phys.* **281**, 40–45. 10.1016/S0301-0104(02)00376-2 (2002).

[17] Hamdi, O., Mighri, F. & Rodrigue, D. Piezoelectric polymer films: synthesis, applications, and modeling. *Polymer Nanocomposite-Based Smart Materials*, (Eds. Bouhfid R., Qaiss A.K. & Jawaid M.) 79–101Woodhead Publishing Series in Composites Science and Engineering, Elsevier, (2020). 10.1016/B978-0-08-103013-4.00005-4

[18] Yang, Y., Zeng, S., Li, X., Hu, Z. & Zheng, J. Ultrahigh and tunable electromagnetic interference shielding performance of PVDF composite induced by nano-micro cellular structure. *Polymers* **14**, 234. 10.3390/polym14020234 (2022). 35054643 10.3390/polym14020234 PMC8781995

[19] Dassan, B. E., Ab Rahman, A. A., Abidin, M. & Akil, H. Carbon nanotube–reinforced polymer composite for electromagnetic interference application: A review. *Nanatechnol. Reviews*. **9**, 768–788. 10.1515/ntrev-2020-0064 (2020).

[20] Galstian, I. Y. at al. Influence of multi-walled carbon nanotubes in polytetrafluoroethylene on the parameters of electronic structure and absorption of ultrahigh-frequency radiation. *Applied Nanoscience* 13, 4977–4987 (2023). 10.1007/s13204-022-02659-4

[21] Lisunova, M. O., Mamunya, Y. P., Lebovka, N. I. & Melezhyk, A. V. Percolation behaviour of ultrahigh molecular weight polyethylene/multi-walled carbon nanotubes composites. *Eur. Polymer J.* **43**, 949–958. 10.1016/j.eurpolymj.2006.12.015 (2007).

[22] Arshad, M. A. & Maaroufi, A. K. Kinetics of dynamic percolation in polymer/carbon composites. *Polym. Eng. Sci.* **60**, 423–433. 10.1002/pen.25298 (2020).

[23] Lebovka, N., Lisunova, M., Mamunya, Y. P. & Vygornitskii, N. Scaling in percolation behaviour in conductive–insulating composites with particles of different size. *J. Phys. D: Appl. Physics*. **39**, 2264–2271. 10.1088/0022-3727/39/10/040 (2006).

[24] Lux, F. Models proposed to explain the electrical conductivity of mixtures of conductive and insulating materials. *J. Mater. Sci.* **28**, 285–301. 10.1007/BF00357799 (1993).

[25] Nishchenko, M. M., Galstian, I. Y., Mykhailova, G. Y., Bozbey, Y. F. & Koda, V. Y. Electrical and Thermoelectric Properties of the Composite Polytetrafluoroethylene, Multi-Walled Carbon Nanotubes. In: Fesenko, O., Yatsenko, L. (eds) *Nanophysics, Nanophotonics, Surface Studies, and Applications. Springer Proceedings in Physics* 183, 523–527 (2016). 10.1007/978-3-319-30737-4_42

[26] Zheng, H. et al. Recent advances of interphases in carbon fiber–reinforced polymer composites: A review. *Compos. Part. B: Eng.* **233**, 109639. 10.1016/j.compositesb.2022.109639 (2022).

[27] Fomenko, I. E. et al. Positron spectroscopy of liquid crystalline organic materials containing С_60_ fullerenes in *Hydrogen Materials Science and Chemistry of Carbon Nanomaterials. NATO Security through Science Series A: Chemistry and Biology.* (Eds. Veziroglu, T.N., 753–756 (2007). 10.1007/978-1-4020-5514-0_94

[28] Ivanenko, K. et al. Influence of Nanofillers Concentration on Physical and Mechanical Characteristics of Their Polymer Composites. In: Fesenko, O., Yatsenko, L. (eds) *Nanomaterials and Nanocomposites, Nanostructure Surfaces, and Their Applications. Springer Proceedings in Physics*, 246, 685–698 (2021). 10.1007/978-3-030-51905-6_46

[29] Zanjanijam, A. R., Bahrami, M. & Hajian, M. Poly(vinyl chloride)/single wall carbon nanotubes composites: investigation of mechanical and thermal characteristics. *J. Vinyl Add. Tech.* **22**, 128–133. 10.1002/vnl.21413 (2016).

[30] Saxena, P. & Shukla, P. A comprehensive review on fundamental properties and applications of poly(vinylidene fluoride) (PVDF). *Adv. Compos. Hybrid. Mater.* **4**, 8–26. 10.1007/s42114-021-00217-0 (2021).

[31] Melezhik, A. V., Sementsov, Y. I. & Yanchenko, V. V. Synthesis of fine carbon nanotubes on coprecipitated metal oxide catalysts. *Russ. J. Appl. Chem.* **78**, 917–923. 10.1007/s11167-005-0420-y (2005).

[32] Soneda, Y. et al. *Carbon*, *Int. Conf., July 14 19*, Lexington, Kentucky, USA, Session 26.1(2001).

[33] Kartel, M., Sementsov, Y., Mahno, S., Trachevskiy, V. & Bo, W. Polymer composites filled with multiwall carbon nanotubes. *Univers. J. Mater. Sci.* **4** (2), 23–31. 10.13189/ujms.2016.040202 (2016).

[34] http://nabivka.com/ru/nanouglerodnie_materiali.html

[35] Tsapko, E. A. & Galstian, I. Y. Positron spectroscopy study of structural defects and electronic properties of carbon nanotubes. *Progress Phys. Met.* **21**, 153–179. 10.15407/ufm.21.02.153 (2020).

[36] Smolnik, S. V. et al. Effect of caesium adsorption on emission properties of (100) and (110) surfaces of tungsten and molybdenum single crystals. Preprint at Research Square (2024). 10.21203/rs.3.rs-5574421/v1

[37] Galstian, I. Y. et al. Low-temperature thermionic converters based on metal nanostructured carbon composites. *Metallofiz Noveishie Tekhnol.* **42**, 451–470. 10.15407/mfint.42.04.0451 (2020).

[38] Chegodaev, D. D., Naumova, Z. K., Dunaevskaya, T. S. & Fluoroplastics (L.: Goskhimizdat, (1960).

[39] Briskman, B. A., Rogova, V. N., Dudarev, V. Y. & Noyfekh, A. I. Study of the crystallinity of polytetrafluoroethylene by X-ray structural analysis and differential scanning calorimetry. *High-molecular Compd.* **31B**, 539–543 (1989).

[40] Lau, S. F., Suzuki, H. & Wunderlich, B. The thermodynamic properties of polytetrafluoroethylene. *J. Polym. Sci.* **22**, 379–405. 10.1002/pol.1984.180220305 (1984).

[41] Mott, N. F. & Gurney, R. W. *Electronic Processes in Ionic Crystals* (Clarendon, 1940).

[42] Brosseau, C. Modeling the interface between phases in dense polymer–carbon black nanoparticle composites by dielectric spectroscopy: where are we now and what are the opportunities. *Macromol. Ther. Simul.* **33**, 2400009. 10.1002/mats.202400009 (2024).

[43] Garkusha, O. M., Makhno, S. M., Prikhodko, G. P., Sementsov, Y. I. & Cartel, M. T. Structural features and properties of polymeric nanocomposites with low concentrations of fillers. *Chem. Phys. Technol. Surf.* **1**, 103–110 (2010).

