# Supplementary material for: Thermophotoinduced electron emission from conductive composite based on polytetrafluoroethylene with carbon nanotubes
Source: Sci Rep. 2025 Aug 14;15:29886. doi: 10.1038/s41598-025-12418-4 (PMC12354696; doi:10.1038/s41598-025-12418-4)

APPENDIX

Some characteristics of composites in the system PTFE - purified CNT and PTFE - purified and dispersed CNT

System PTFE + purified MWCNT	Density of composite powders compressed at 380^0C, g/cm^3	System PTFE + purified and dispersed MWCNT	Density of composite powders compressed at 380^0C, g/cm^3
PTFE (pure)	2,1470, 2,1379	PTFE + 0,05%(мас.)	2,1554
PTFE + 0,05 %wt	2,1573	PTFE + 0,1 %wt	2,1498
PTFE + 0,1 %wt	2,1582	PTFE + 0,5 %wt	2,1482
PTFE + 0,5 %wt	2,1530	PTFE + 1,0 %wt	2,1457
PTFE + 1,0 %wt	2,1426	PTFE + 2,0 %wt	2,1578
PTFE + 2,0 %wt	2,1369	PTFE + 3,0 %wt	2,1531
PTFE + 3,0 %wt	2,1247	PTFE + 5,0 %wt	2.1464
PTFE + 5,0 %wt	2,1035	PTFE + 10,0 %wt.	2,1249
PTFE + 15,0 %wt	2,0158	PTFE + 15,0 %wt	2,2007
PTFE + 20,0 %wt	1,8775	PTFE + 20,0 %wt	2,0174

Distribution of Nanotubes by Outer Diameter

The figure shows the distribution of the number N of nanotubes as a function of the outer diameter. It can be seen that it is in the range of 7-25 nm with a maximum of 11 nm. Most of the tubes (at the 0.5 level) have a diameter of 10-15 nm. The thickness of the CNT walls varies within 2-11 nm, with the maximum distribution between 3 and 4 nm (Fig.) The number of hexagonal layers in the nanotubes was within 5-17 carbon layers, and the most probable value is 9 carbon layers. The total length of the 25 mg CNT with the given parameters was calculated to be 280 thousand km.

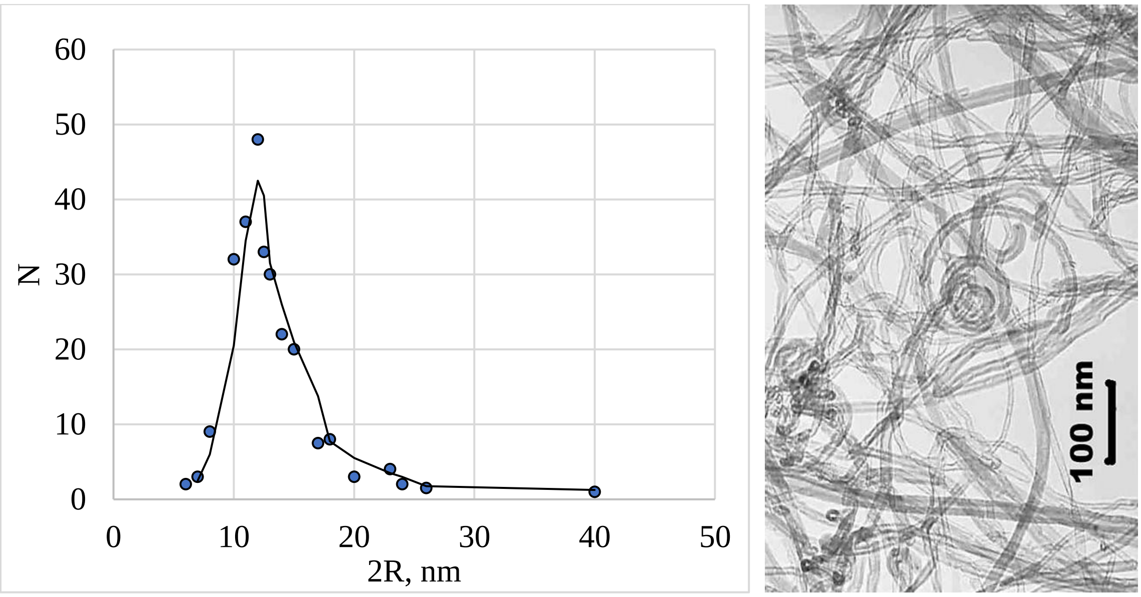

Fig. Statistical distribution of nanotubes by outer diameter

The TEM image clearly shows multilayer carbon nanotubes. In accordance with the basic assumption of transmission electron microscopy that the intensity of the image contrast is

directly dependent on the amount of scattering material, i.e. darker areas on positive prints correspond to thicker areas of the sample or heavier atoms present in the sample, the observed narrow dark bands are attributed to the walls of the nanotube.

Resistance (two-contact) PTFE+10 wt% CNT, Ohm

	longitudinal	transverse
	312	170
	297	210
	144	205
	350	163
	133	160
	283	241
	290	232
	172	250
	237	235
	188	123
	224	138
	243	
	212	
	129	
	214	
	168	
	191	
	220	
	165	
	139	
average	215,55	193,36

Laser-stimulated emission research

The research of emission properties was carried out on the installation, the scheme of which is shown in Fig.

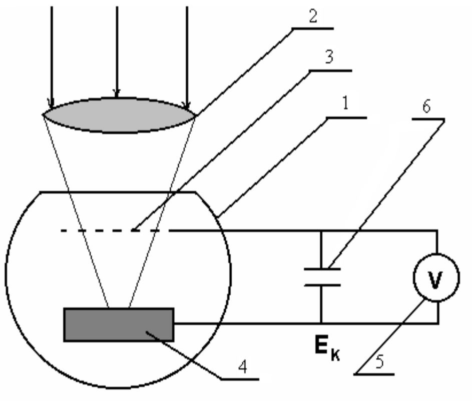

Fig. Scheme of the installation for the study of laser-stimulated emission: 1- Vacuum chamber; 2- Focusing lens; 3- Anode; 4- Cathode; 5- Voltmeter; 6- Capacitor.

A vacuum of p~10^{-1} - 10^{-2} Pa was created in a sealed chamber, a cathode and an anode Mo grid were placed in this chamber. The electrode outputs were connected to a capacitor, which was charged with an emission current.
Radiation was introduced into the vacuum chamber through a window made of quartz glass, which is transparent in the optical range, including the near ultraviolet spectrum. The cathode was heated from the anode side, made of molybdenum wire 0.15 mm thick and located at a distance of 1,5 mm from the cathode surface, by pulses of a YAG: Nd laser (λ=1,06 μm).
At certain values of laser pulse energy, electron emission starts from the cathode surface. After overcoming the vacuum space, the electrons fall onto the anode and from there onto the capacitor. The criterion for comparison of the emission characteristics was the energy of the beginning of emission (the minimum energy of the laser pulse (l.i.) at which the increase of the voltage on the capacitor is recorded) and the value of the voltage on the capacitor at a certain value of the l.i. energy.
The samples under study served as emitters, and the anode material remained unchanged in all studies. A capacitor with a capacitance of C=10 μF was connected in parallel with the electrodes. When the target (emitter) was heated by laser pulses with a duration in the range of 1-3.7 ms and a radiation wavelength of λ=1.06 μm with an energy in the pulse that gradually changed from minimum to maximum values for each of the samples, an emission current was generated that charged the capacitor.

PTFE - 5% CNT

UL	EL, Дж	Uc, мВ	Uc, мВ	Uc, мВ	Ср.знач	j, А/см^2
260	0.0396	0	0	0	0	0
280	0.0866	0.2	0.3	0	0.166666667	0.000268
300	0.175	3	2.7	2.6	2.766666667	0.004449
320	0.2566	9.2	6.8	5.1	7.033333333	0.011311
340	0.3	14.2	9.9	8.4	10.83333333	0.017423
360	0.4233	15.3	13.2	10.3	12.93333333	0.0208
380	0.5383	19	16.2	17.4	17.53333333	0.028198
400	0.64	32.1	31.1	25.9	29.7	0.047765
420	0.77	58.2	58.1	54.4	56.9	0.091509
440	0.9333	86	74.5	69.3	76.6	0.123191
460	1.08	123	122.2	125.3	123.5	0.198617

PTFE - 10% CNT

UL	EL, Дж	Uc, мВ	Uc, мВ	Uc, мВ	Ср.знач	j, А/см^2
260	0.0396	0.1	0	0.1	0.066666667	0.000107
280	0.0866	1.5	1.3	1.4	1.4	0.002252
300	0.175	6.5	6.2	6	6.233333333	0.010025
320	0.2566	12.6	9.4	9	10.33333333	0.016618
340	0.3	6.2	0.9	5.7	4.266666667	0.006862
360	0.4233	5.3	7.3	13.5	8.7	0.013992
380	0.5383	16.9	18.5	20.7	18.7	0.030074
400	0.64	12.6	47.6	65	41.73333333	0.067117
420	0.77	102	86	86	91.33333333	0.146886
440	0.9333	125	110	130	121.6666667	0.195669
460	1.08	195	184	186	188.3333333	0.302885

Vacuum chamber for studying the emission properties of cathodes under the influence of concentrated radiation

The vacuum chamber (VC) is designed to record the current-voltage characteristics (IVC) of cathodes during their irradiation with concentrated solar radiation. The VC provides operation in two modes: in the mode of natural concentrated solar radiation, when the VC can be oriented using a special system in such a way that the cathode is in the focus of the mirror, which ensures its high temperature.
The second mode provides operation of the VC under conditions of irradiation of the cathode with a high-pressure xenon lamp using a focusing system.
The VC is designed to operate at a pressure of 0,5 to 10^{-3} torr, which is provided by specially designed fittings.
The VC is the 4th generation of advanced cathode mounting and anode heat dissipation systems. In this development, the cathode is mounted on a low thermal conductivity ceramic, and the anode is mounted on a thick copper plate, which transfers heat to a copper flange, the outer diameter of which is outside the chamber, and to which a massive aluminum radiator is screwed. The VC electrical measurement system allows the current and voltage of the cathode-anode, the temperatures of the cathode and anode, and the pressure in the chamber to be recorded simultaneously on a hard disk. Idle and short-circuit measurements are also provided. To ensure emission only from the emitter, it is necessary to develop an anode cooling system.

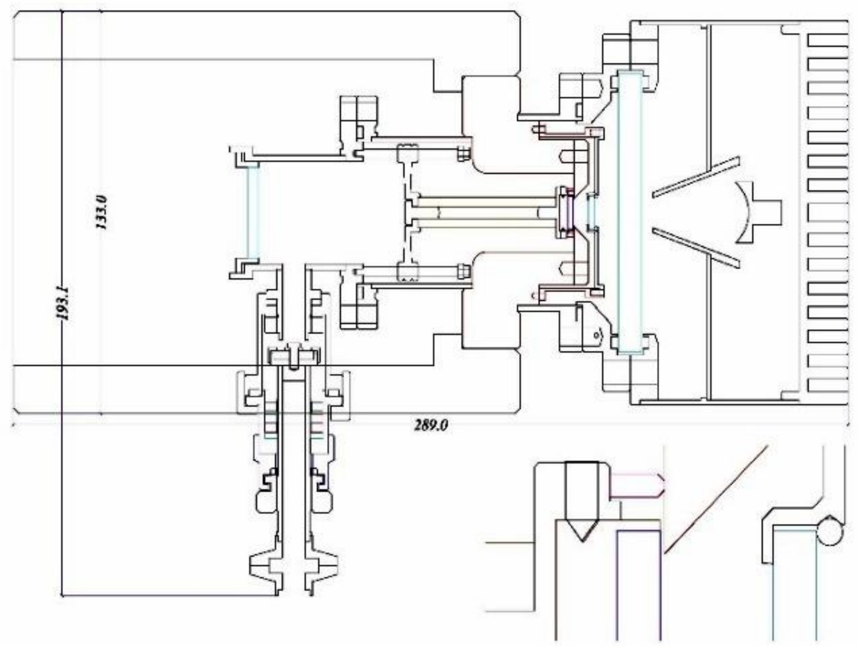

Scheme of a vacuum chamber for studying the effect of concentrated solar radiation on the emission properties of cathodes

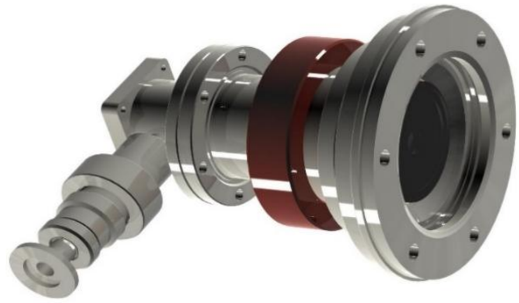

View of a chamber used to study the effect of concentrated solar radiation on the emission characteristics of cathodes (without radiator)

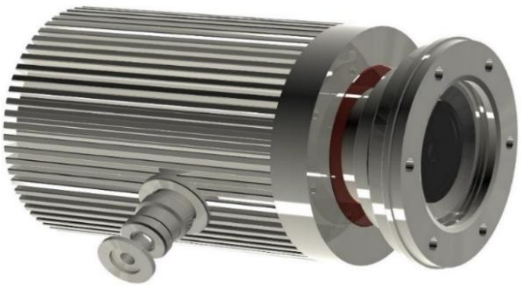

View of a chamber used to study the effect of concentrated solar radiation on the emission characteristics of cathodes (with radiator)

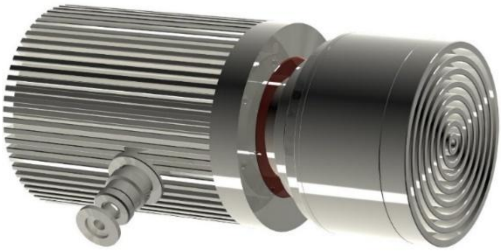

General view of a chamber for studying the effect of artificial solar radiation on the emission characteristics of cathodes (with radiator)

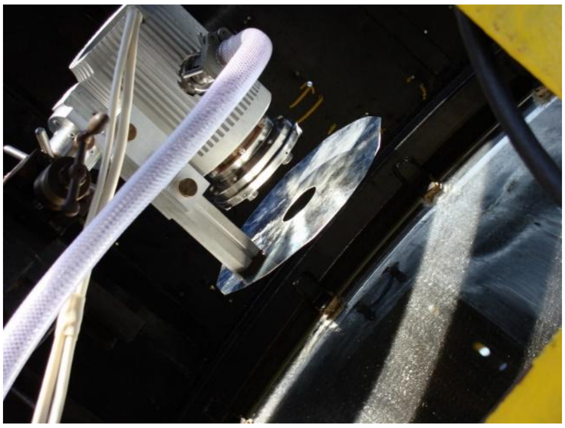

Mount the vacuum chamber and align it relative to the focus of the mirror (the edge of the focusing mirror is visible in the upper left of the image).

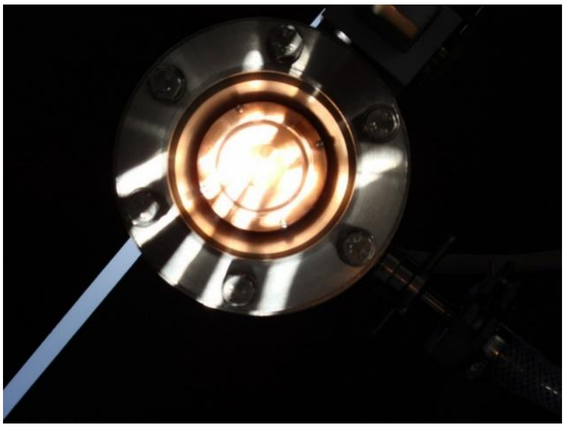

Vacuum chamber for studying the emission power during the study (view from the side of the incident solar radiation).

The emitter was heated by pulsed laser or continuous light radiation passing through a wire anode. The optical radiation was focused on the surface of the cathode in the form of a spot 10

mm in diameter. In this case, the cathode temperature, measured by a chromel-alumel thermocouple, was set in the temperature range of 50-500 ˚C.

Calculation of the angular correlation spectra of annihilation radiation (ACAR)

ACAR were measured in the angular range from -35 to 25 mrad using an annihilation spectrometer with a long-slit geometry and an angular resolution of 1,07 mrad. The spectra were approximated by the least-squares method by the sum of the parabolic contribution (PC) and two Gaussians - wide (WG) and average (MG) with variances σ_b, and σ_i, respectively:

$$I(\Theta) = I_p(\Theta) + \sum_{j=1}^{2} I_G^j(\Theta)$$

The angular broadening spectra of annihilation photons ASAP were used to measure the electron momentum distribution (in reciprocal space), which was used to calculate the distribution of the electron wave functions (in real space). The quantitative (inversely proportional) relationship between the width of the Gaussian components of the ASAP spectra and the sizes of the anions or the width of the interatomic (intermolecular) gaps allows to obtain information about the characteristics of the internal structure of nanostructural elements (nanotubes, nanopores) of the system. Thus, it was possible to determine the size of atoms or defects that trap positrons, or areas in nanostructures with reduced atomic density into which positrons fall under the action of the Coulomb field. A parabola and two Gaussians were obtained from the decomposition of the ASAP spectra. The probabilities of positron annihilation processes with free electrons ($P_{\Theta F}$), with π-electrons between carbon layers rolled into cylinders (S_1), and with localized electrons of broken covalent bonds in defects of the hexagonal layer (S_2) were calculated from the area under the parabolic contribution curve and the Gaussians.

Electron microscope images of the PTFE+10 wt.% CNTs composite

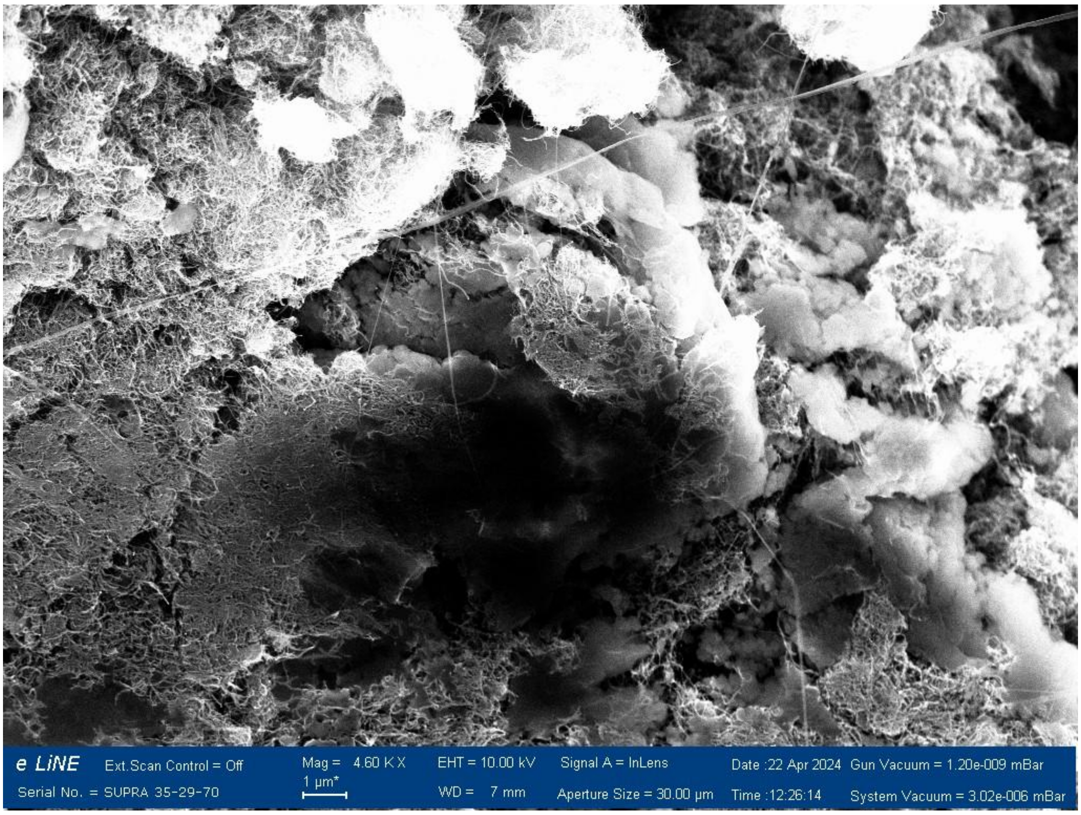

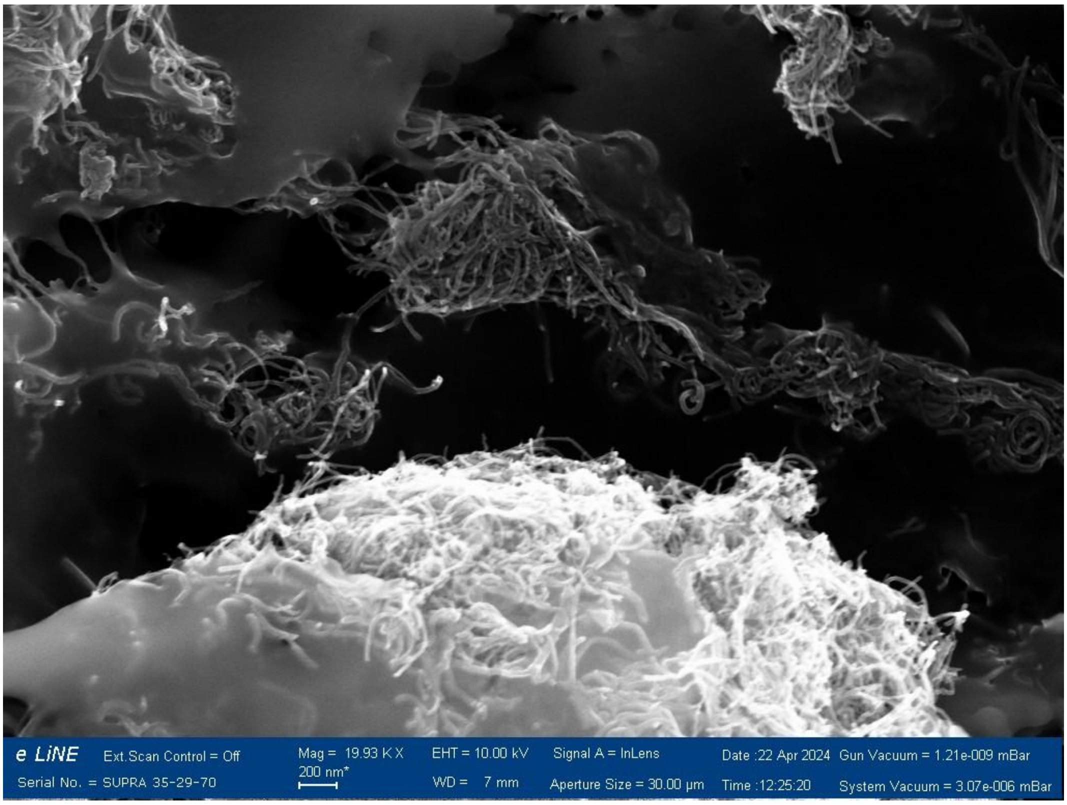
e LiNE
Ext.Scan Control = Off
Serial No. = SUPRA 35-29-70
Mag = 19.93 K X
200 nm*
EHT = 10.00 kV
WD = 7 mm
Signal A = InLens
Aperture Size = 30.00 µm
Date :22 Apr 2024
Time :12:25:20
Gun Vacuum = 1.21e-009 mBar
System Vacuum = 3.07e-006 mBar

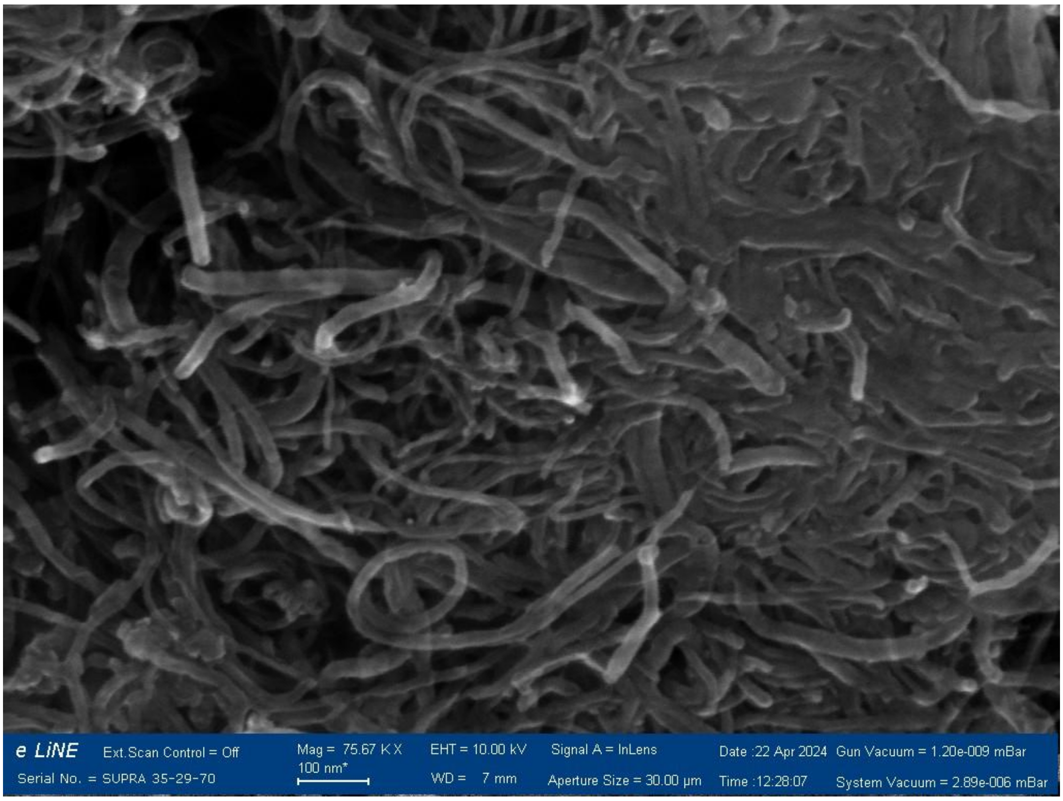
e LiNE
Ext.Scan Control = Off
Serial No. = SUPRA 35-29-70
Mag = 75.67 K X
100 nm*
EHT = 10.00 kV
WD = 7 mm
Signal A = InLens
Aperture Size = 30.00 µm
Date :22 Apr 2024
Time :12:28:07
Gun Vacuum = 1.20e-009 mBar
System Vacuum = 2.89e-006 mBar

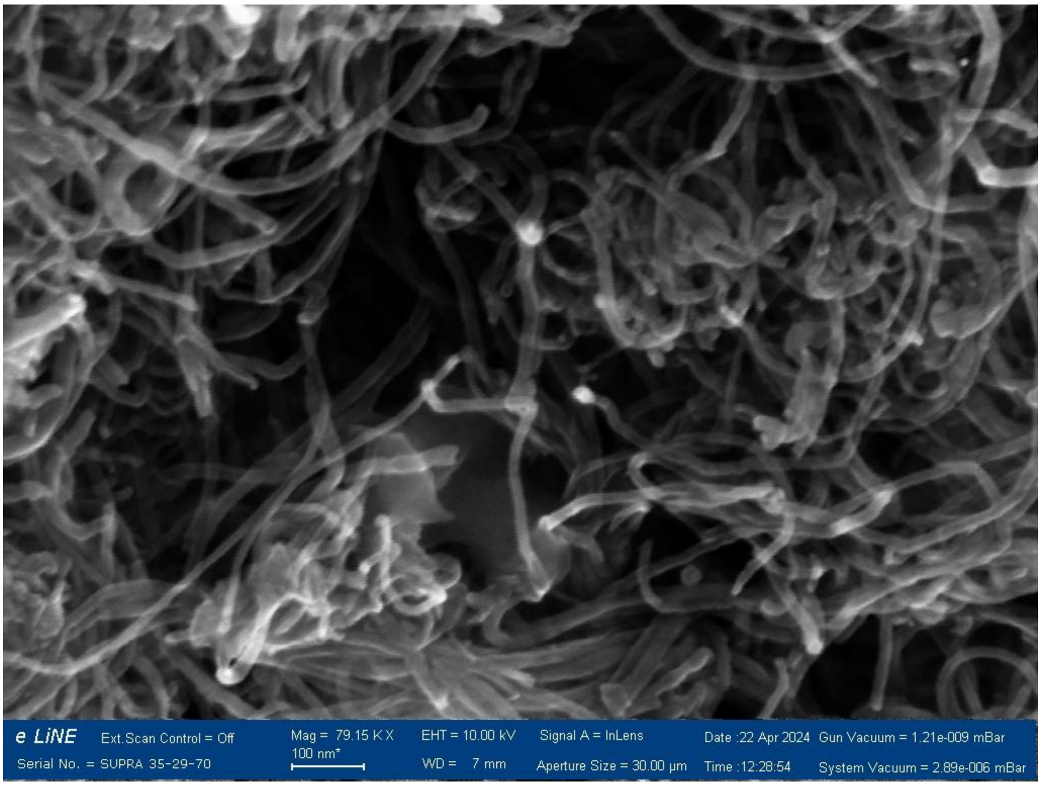

Electron microscope images of the PTFE

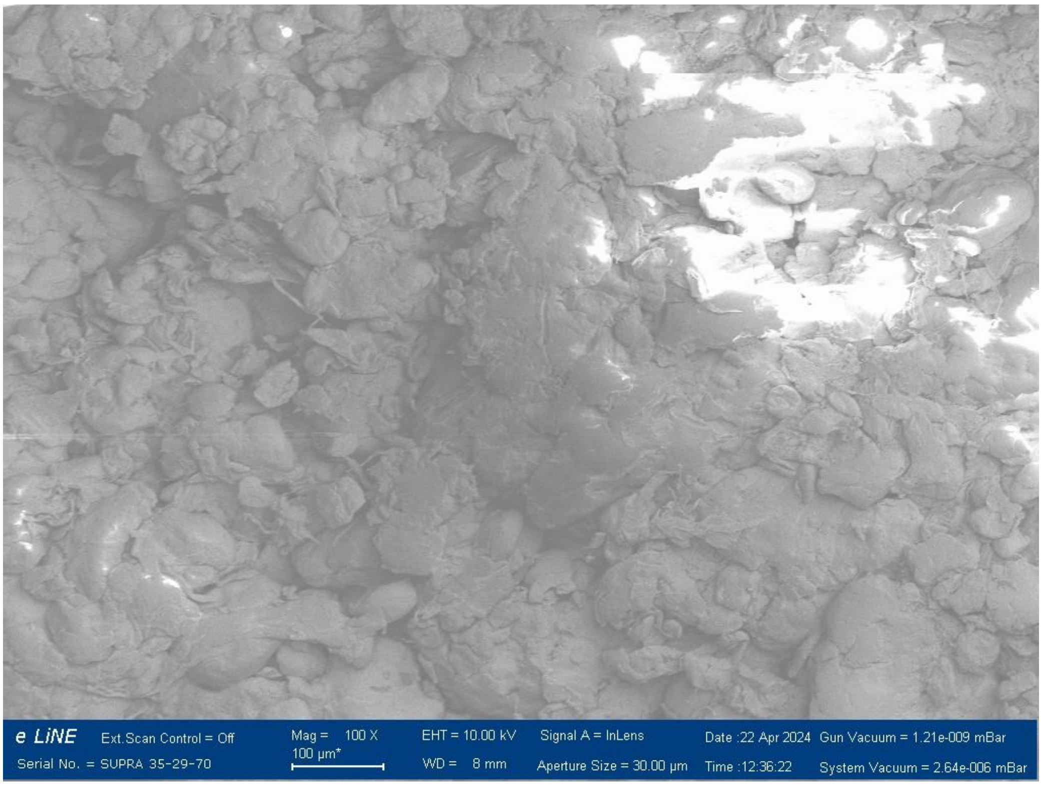

Supplement: Supplementary file 1 — Supplementary Material 1 [file 41598_2025_12418_MOESM1_ESM.pdf]
