# Supplementary material for: Thermophotoinduced electron emission from conductive composite based on polytetrafluoroethylene with carbon nanotubes
Source: Sci Rep. 2025 Aug 14;15:29886. doi: 10.1038/s41598-025-12418-4 (PMC12354696; doi:10.1038/s41598-025-12418-4)
Supplement: Supplementary file 2 — Supplementary Material 2 [file 41598_2025_12418_MOESM2_ESM.docx]

**Statements and Declarations**

**Financial support:**

This work was supported by Project No. 0123U102275 of the National Academy of Sciences of Ukraine and Volkswagen Foundation UKRATOP Program.

**Competing interests:**

The authors have no competing interests relevant to the content of this article.

**Compliance with ethical standards:**

This work does not include studies involving human and/or animal participants.

**Informed consent:**

All listed authors have approved the manuscript prior to submission, including the names and order of authors.

**Data availability statements:**

All data collected or analyzed during this study are included in this article and its supplemental information files.
