# Supplementary material for: Thermophotoinduced electron emission from conductive composite based on polytetrafluoroethylene with carbon nanotubes
Source: Sci Rep. 2025 Aug 14;15:29886. doi: 10.1038/s41598-025-12418-4 (PMC12354696; doi:10.1038/s41598-025-12418-4)
Supplement: Supplementary file 3 — Supplementary Material 3 [file 41598_2025_12418_MOESM3_ESM.docx]

Author Contributions

Iryna Galstian (corresponding author) https://orcid.org/0000-0002-5633-0602 Leibniz Institute for Solid State and Materials Research, Dresden, Germany; [i.galstian@ifw-dresden.de](mailto:i.galstian@ifw-dresden.de)

Evgen Len https://orcid.org/0000-0002-1554-2342 G.V. Kurdyumov Institute for Metal Physics of the N.A.S. of Ukraine, Kyiv, Ukraine; [len.evgeniy@gmail.com](mailto:len.evgeniy@gmail.com)

Mykola Shevchenko G.V. Kurdyumov Institute for Metal Physics of the N.A.S. of Ukraine, Kyiv, Ukraine; [elenya69@ukr.net](mailto:elenya69@ukr.net)

Yevgen Tsapko G.V. Kurdyumov Institute for Metal Physics of the N.A.S. of Ukraine, Kyiv, Ukraine; [tsapkoe@gmail.com](mailto:tsapkoe@gmail.com)

Tatiana Shatnii https://orcid.org/0000-0002-2018-3604 G.V. Kurdyumov Institute for Metal Physics of the N.A.S. of Ukraine, Kyiv, Ukraine; [t.shatnii@gmail.com](mailto:t.shatnii@gmail.com)

Oksana Lisova https://orcid.org/0000-0002-9605-8420 Chuiko Institute of Surface Chemistry of the N.A.S. of Ukraine, Kyiv, Ukraine; [oksana.garkusha@gmail.com](mailto:oksana.garkusha@gmail.com)

All authors contributed fully to this study.

Conceptualization: Iryna Galstian, Evgen Len

Methodology: Mykola Shevchenko, Evgen Len, Iryna Galstian

Analysis and investigation: Mykola Shevchenko, Iryna Galstian, Evgen Len, Yevgen Tsapko, Oksana Lisova, Tatiana Shatnii

Writing - original drafting: Iryna Galstian, Evgen Len, Tatiana Shatnii;

Writing - review and editing: Iryna Galstian, Evgen Len;

Funding acquisition: Iryna Galstian, Evgen Len;

Supervision: Evgen Len

All authors have read and approved the final manuscript
